# Vertical Dimension Control in Two Different Treatment Protocols: Invisalign First and Bite Block—A Retrospective Study

**DOI:** 10.3390/children11101252

**Published:** 2024-10-17

**Authors:** Giuseppina Laganà, Arianna Malara, Daniel Palmacci, Patrizio Bollero, Paola Cozza

**Affiliations:** 1 Department of Health Sciences, Unicamillus—Saint Camillus International University of Health Sciences, Via di Sant’Alessandro, 8, 00131 Rome, Italypaola.cozza@unicamillus.org (P.C.); 2 Department of Systems Medicine, University of Rome “Tor Vergata”, Viale Oxford 81, 00133 Rome, Italy

**Keywords:** clear aligner, Invisalign first, bite block, vertical dimension, growing patient, quad-helix

## Abstract

Background: The aim of the present study was to compare the vertical dimension changes, before and after treatment, in two groups of growing patients, one group treated with clear aligner therapy versus a group treated with Quad-helix and bite-block therapy. Methods: The studied sample was composed of n. 40 patients (20 females and 20 males with a mean age of 8.6 ± 1.8 years), enrolled from the Department of Orthodontics at Policlinico of Rome Tor Vergata. The original sample was randomly divided into two groups: Group IF (Invisalign First) and Group BB (Quad-helix and bite block). Pre- (T0) and post-treatment (T1 after 12 months) lateral cephalograms were collected from all the selected patients. Nine cephalometric parameters, both angular and linear, were measured and recorded for each cephalogram. Results: No statistically significant changes were found between both the IF and BB groups at T0, while statistically significant changes were observed in both groups (BB and IF) between T0 and T1 (after 12 months of active therapy), *p* < 0.005. Conclusions: Both therapies were able to control the patient’s vertical condition. To date, the use of conventional appliances seems to have slightly better efficacy in controlling the vertical dimension than aligner therapy.

## 1. Introduction

As is well known, malocclusions can affect subjects in all three planes of space. Traditionally, the orthodontist usually focuses on antero-posterior dentoskeletal relationships, but several malocclusions are the result of an abnormal vertical growth pattern.

The patient’s altered vertical dimension is certainly one of the most challenging problems to manage in the orthodontic field [1,2]. Indeed, very often, the goal of treatment is to maintain or reduce the patient’s vertical dimension, especially in hyperdivergent subjects characterized by a high mandibular plane angle and a long face. Long face syndrome is formed by the excessive vertical growth of the face with a backwardly rotated mandible, an increased lower face height and a tendency of open bite in severe cases [3,4]. The success of a treatment is due to the orthodontist’s capacity to control vertical tooth movements, since posterior tooth extrusion is the main etiology of undesirable side effects, such as mandibular backward rotation [1,5]. A bite block (BB) showed positive therapeutic outcomes in individuals with a tendency for dentoskeletal open bite, as it minimized posterior tooth extrusion, and it allowed mandibular counterclockwise rotation to obtain bite closure [6,7,8]. The favorable results of BB therapy to control the direction of mandibular growth have been noticed in experimental studies in both animals and humans [9].

Over the past few years, orthodontists have started utilizing aligners for the treatment of malocclusions thanks to their esthetics, efficacy and comfort [10,11]. As fixed appliances, clear aligners were effective in treating malocclusion and allowed for improved oral hygiene, a lower risk of tooth decay [12], the segmented movement of teeth and the shortening of treatment duration [13]. One of the potential benefits of clear aligners is the “bite-block effect”. It is evident that the position of two thermoplastic aligners on the dental arches increases the vertical dimension, modifying the quantity and quality of the occlusal contacts [14]. Clear aligners are generally about 0.6 mm thick [15], and so almost 1 mm of material is positioned between the arches. The thickness of the aligner material in combination with occlusal forces results in the intrusion and/or extrusion resistance of the posterior teeth during treatment [16].

The purpose of the current retrospective investigation was to evaluate the modification of the vertical dimension using cephalometric analysis in growing patients undergoing two different treatments: clear aligners vs. bite blocks. This is certainly a groundbreaking study, as it is the first to make this comparison. The null hypothesis is of no discrepancy between the groups subjected to different treatments.

## 2. Materials and Methods

This study followed the principles laid down by the World Medical Assembly in the Declaration of Helsinki 2008 on medical protocols and ethics, and it was approved by the Ethical Committee of the University of Rome Tor Vergata (protocol number: 33.23). Written consent was obtained from the parents of all the subjects included in the study.

The sample was composed of n. 40 patients (20 females and 20 males with a mean age of 8.6 ± 1.8 years) enrolled from the Orthodontics Department at the University of Rome Tor Vergata, Italy (Table 1). The original sample was divided into the following two groups depending on the type of treatment:

- Group IF (Invisalign First therapy), composed of 20 patients (10 females and 10 males with a mean age of 8.7 ± 2.1).
- Group BB (Quad-helix and bite block), composed of 20 patients (10 females and 10 males with a mean age of 8.5 ± 1.5).

**Figure 1:**
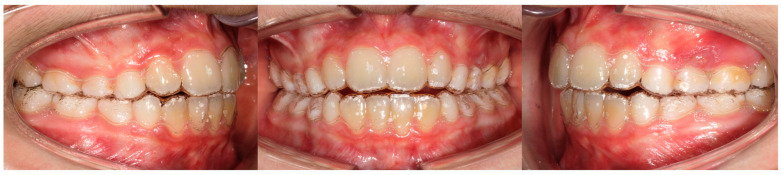
Invisalign appliance.

The IF group was treated with the Invisalign First system (Figure 1). This system uses impressions or intraoral scans which are converted through stereolithographic technology (.stl) into virtual models and then launched with ClinCheck software Pro 6.0, a three-dimensional modeling program that allows for a virtual simulation of teeth movements. A series of aligners is then produced to gain the needed corrections [17].

**Figure 2:**
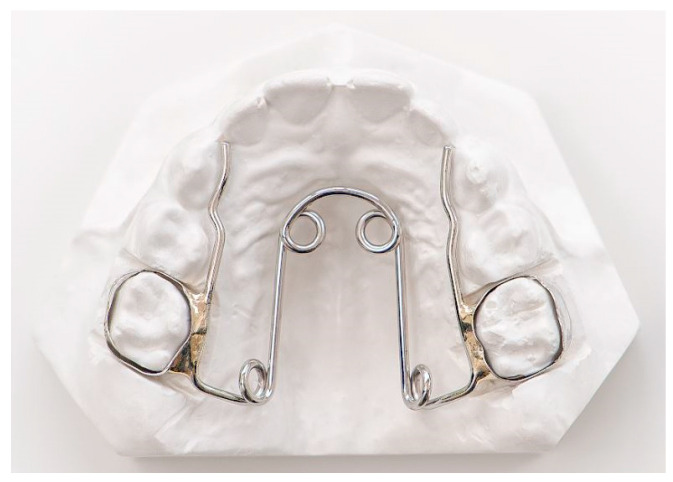
Quad-helix (orthodontic device made by Orthosystem Roma s.r.l., Roma, Italy).

**Figure 3:**
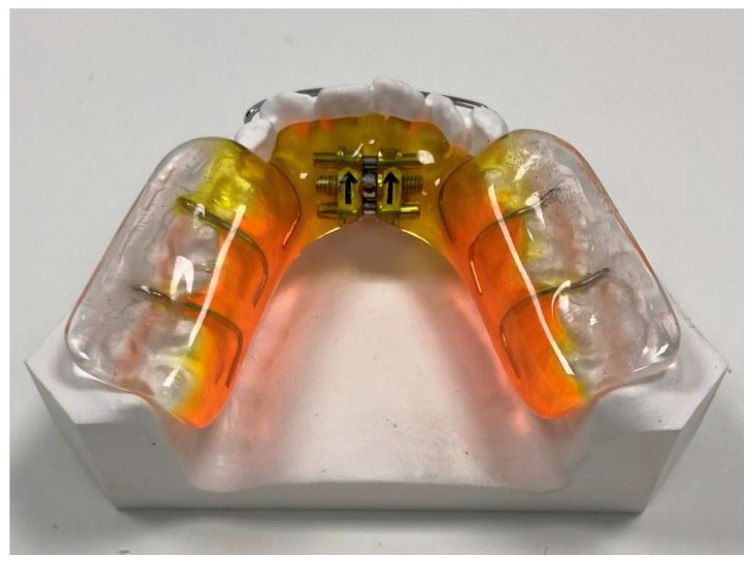
Bite block (orthodontic device made by Orthosystem Roma s.r.l, Roma, Italy).

The BB group was treated with Quad-helix (QH) and bite-block (BB) therapy. The QH appliance used in this study was made of 0.036 in stainless steel wire soldered to bands on the second deciduous molars or the first permanent molars (Figure 2). The lingual arms of the appliance extended mesially to the deciduous canines or to the permanent incisors. The anterior helices were brought as far forward on the palate as possible. The BB was designed as a Schwarz device for the mandibular arch, with resin splints of 5 mm thickness in the posterior occlusal region (Figure 3). The BB was prescribed for 12 months to control the vertical dimension. Patients were instructed to wear the BB full-time, 22 h a day, except for at meals and while toothbrushing. Both orthodontic devices were made by Orthosystem Roma s.r.l., Roma, Italy.

For both groups, the inclusion criteria were the inter-transitional phase of mixed dentition; good oral hygiene; mild to moderate dentoalveolar crowding, calculated by Little’s Irregularity Index [18]; hyper-divergence (FMA > 28° and SnGoGn > 36°), assessed by cephalometric analysis performed on the lateral cephalogram; and good compliance in wearing the removable appliances. All the patients in an active permutation phase, those with a severe skeletal transverse discrepancy and normo-hypodivergent patients (FMA < 28° and SnGoGn < 36°) were excluded from the study.

Pre- (T0) and post-treatment (T1 = 12 months) lateral cephalograms were collected from all the selected patients, and a cephalometric software (Viewbox, version 4.0, dHAL software, Kifissia, Greece) was used to type the radiographs of each subject. The dental impressions of each patient involved were taken by an intraoral iTero scanner. The .stl files of the patients treated with the traditional technique were sent to the laboratory for the fabrication of the orthodontic devices. For the patients undergoing the Invisalign First treatment, the .stl files were imported in the Clincheck software [19].

### 2.1. Data Measurement

The radiographs were manually traced by the same expert operator (D.P.) blinded about the study. A total of nine cephalometric parameters (seven angular and two linear) were measured and recorded for each cephalogram:

1. **FMA** angle (degrees) between the Frankfort Horizontal (Po-Or) and the mandibular plane (Go-Me);
2. **SN°GoGn** angle between the Sella–Nasion plane and Steiner’s mandibular plane (Gonion–Gnathion);
3. **PF°Poccl** angle between the Frankfort Horizontal (Po-Or) and the occlusal plane;
4. **SN°Poccl** angle between the Sella–Nasion plane and the occlusal plane;
5. **ANS-PNS°Go-Me** angle between the maxillary plane and Tweed’s mandibular plane (Gonion–Menton);
6. **SN°ANS-PNS** angle between the Sella–Nasion plane and the maxillary plane;
7. **ArGo°GoMe** angle between the plane of the mandibular ramus and Tweed’s mandibular plane (Gonion–Menton);
8. **SGo/NMe** ratio of the posterior facial height (the linear distance between Sella and Gonion) and the anterior facial height (the linear distance between Nasion and Menton);
9. **N-ANS/ANS-Me** ratio of the upper anterior facial height (the linear distance between Nasion and ANS) and the lower anterior facial height (the linear distance between ANS and Menton).

To determine the reproducibility of the method, the same cephalometric analysis was re-performed by the same operator (D.P.) ten days later. To compare the two measurements (systematic error), a paired *t*-test was conducted. The magnitude of the random error was determined utilizing the method of the moment’s estimator [20].

### 2.2. Statistical Analysis

The data were collected in Microsoft Excel (version 16.61.1) and elaborated in the Statistical Package for the Social Sciences Windows, version 15.0 (SPSS, Chicago, IL, USA). The qualitative data were analyzed using the Chi-square test of Pearson to determine if the distributions between age, gender and the other variables were statistically different. The *p* value for statistical significance was set at 0.05, so any value less than *p* < 0.05 was interpreted as statistically significant.

Descriptive statistics were used to describe both sample groups (IF and BB) in terms of age and sex. The power of the study for the independent sample *t*-test was estimated from the sample size of the two groups and an effect size equal to 0.9 [20]. The power was 0.80 at an alpha level of 0.05 (SigmaStat 3.5, Systat Software, Point Richmond, CA, USA). As the data were normally distributed, a paired *t*-test was chosen to confront the T1–T0 variations. The level of significance was set at 5%. The software used to analyze the data was the SPSS (Statistical Package for the Social Sciences), version 18.0 (IBM Corp, Chicago, IL, USA).

## 3. Results

Among the multiple digital measurements, no systematic error was observed. This was made possible by the accurate definitions of points and by a previously trained experienced examiner. No statistically significant changes were found between both the IF and BB groups at time T0 (Table 2) and at time T1 (Table 3), while statistically significant changes were observed in both groups (BB and IF) between T0 and T1 (after 12 months of active therapy).

In the group of patients treated with Quad-helix and bite-block therapy (BB group), eight values were modified (Table 4). It was found that at time T1, the most radically changing parameters were SN°GoGn (*p* = 0.0007), SN°Poccl (*p* = 0.0001), N-ANS/ANS-Me (*p* = 0.005) and ArGo°GoMe (*p* = 0.0001), thus showing a general tendency for ante-rotation of the skeletal bases and of the occlusal plane. The only measurement which did not show statistically significant differences was FMA (*p* = 0.0973); in fact, it remained stable in the post-treatment.

In the second group (IF), only seven values were changed (Table 5), including skeletal divergence and growth projection in the Invisalign First group, which improved considerably after one year of treatment—FMA (*p* = 0.0009) and SNGoGn (*p* = 0.0002)—while the occlusal plane PF°Poccl (*p* = 0.318) and the orientation of the maxillary plane SN°ANS-PNS (*p* = 0.393), in relation to the skull bases, did not seem to change significantly.

Therefore, it appeared that, in the BB group, the tendency for ante-rotation of the structural and of the dental components was slightly greater than that in the IF group.

## 4. Discussion

The main objective of the current study was to evaluate the vertical dimension variations using cephalometric analysis in growing patients undergoing two different treatments: clear aligners vs. Quad-helix and bite-block therapy. This is definitely an innovative study, since it is the first to make this comparison. Regarding the vertical condition, the results showed that in both groups (IF and BB) there was an improvement in cephalometric variables, which was slightly more significant in some bite-block-group variables.

These findings corresponded to other studies; however, as is well known, investigations evaluating the extent of vertical correction with clear aligners are very few and recent. In 2020, Harris et al. [21] showed that the amount of vertical correction was directly related to the digital planning of the ClinCheck (supported by an excellent degree of patient collaboration) involving anterior sector extrusion and molar intrusion. This “bite-block effect” was already reported with aligners and was ascribed to the thickness of material covering the posterior teeth and to the biting force exercised by patients [22].

A retrospective study assessing the efficacy of clear aligners for vertical dimension control in deep and open-bite cases stated that the main correction mechanism for an open bite came by applying incisor extrusion with an average value of 1.5 mm. No major conclusions could be drawn, in this work, for the treatment of open bites, because of the small size of the sample [23]. Conversely, a different retrospective paper analyzed 30 cases of adult open bite utilizing cephalometric analysis, and it reported that the correction of the open bite was mainly through the counterclockwise rotation of the mandible due to lower molar intrusion [24].

Garnett et al. demonstrated that clear aligners can be successful in the control of the vertical dimension and in the correction of severe anterior open bites in adult hyperdivergent patients without using TADs or other auxiliaries [25].

In a 2022 case report [26], the authors examined the skeletal divergence of a hyperdivergent adult patient with open-bite malocclusion, treated with clear aligners. The results of the study showed that after one year of treatment, not only was a molar intrusion of 1.5 mm realized, but many cephalometric parameters benefited: increased Wits, improved sagittal projection of the chin symphysis, and others. The limitations of aligner studies are often related to the lack of growing patient samples, which is the main feature of the current investigation. In a 2022 study, Standerini et al. [27] demonstrated that aligners were able to significantly influence the orientation of the occlusal plane, and thus altered tooth extrusion or intrusion in the growing patients and in the teenagers. In particular, in cases of deep bite, a good programming of the ClinCheck treatment plan allowed for the correction of the curve of Spee intruding the incisors and extruding the latero-medial sectors, therefore resolving crowding while modifying the vertical projection.

Regarding bite-block therapy, in a less recent paper, published by Iscan et al. [28], a group of growing patients who underwent bite-block therapy was examined and compared with a control group matched for age and type of malocclusion. After an evaluation of about nine months, the results showed that in the group treated with bite blocks (with a thickness of about 10 mm), a much more pronounced tendency towards mandibular antero-rotation was found in the treated group than in the untreated one, which instead maintained a tendency towards mandibular post-rotation. Already in 1992, Iscan [29] published another study analyzing the influence of two different types of bite blocks on facial morphology in a growing patient. It showed that even without statistically significant differences among the two groups, a reduction in skeletal divergence correlated closely with a reorientation of the mandibular ramus inclination. Regarding Quad-helix therapy, the mechanism of action was already known in 2007 through the study by Cozza et al., which aimed to analyze the outcomes of QH therapy in growing subjects with dentoskeletal open bite and thumb-sucking habits, comparing them with a control group of untreated subjects with similar baseline vertical relationships. The QH therapy produced a clinically significant 1.8° posterior rotation of the palatal plane [30].

Another study showed that QH treatment in mixed-dentition patients with maxillary incisor crowding gave rise to spontaneous distal tipping and impeded vertical eruption of the maxillary second molars with distalization and impeded the extrusion of the maxillary first molars [31].

Lastly, some limitations of the present study should be considered. Certainly, the small study sample needs to be expanded. In addition, this study analyzed only facial divergence control and not dentoalveolar expansion, which may be a source of future investigations.

## 5. Conclusions

Both conventional therapy (Quad-helix and bite block) and aligner therapy were able to control the patients’ vertical condition. To date, the use of conventional appliances seems to have a slightly better efficacy on controlling the vertical dimension than aligner therapy. The null hypothesis is thus confirmed.

Further studies are needed, however, to understand the influence that aligners have on growing patients.

## Figures and Tables

**Table 1:** Descriptive analysis of the sample.

		Group IF	Group BB
**N.**		20	20
**Gender**	**M**	10	10
	**F**	10	10
**Mean age**		8.7 ± 2.1	8.5 ± 1.5
**Time of treatment**		12 months	12 months

**Table 2:** Descriptive statistics and statistical comparison between IF and BB groups at time T0.

	BB	IF	*p* Value
**FMA**	34° ± 1.3°	31° ± 0.9°	ns
**SN°GoGN**	44° ± 2.1°	38° ± 1.9°	ns
**Poccl ° PF**	16° ± 0.7°	11° ± 0.6°	ns
**N-ANS/N-SMe**	42% ± 1.3%	43% ± 1.7%	ns
**Sn°ANS-PNS**	9° ± 0.5°	9° ± 0.3°	ns
**Sn°Poccl**	26° ± 1.4°	21° ± 1.2°	ns
**SGo/N-Me**	57° ± 1.3 *	59° ± 0.7°	ns
**ArGo/GoMe**	136° ± 2.5°	135° ± 2.2°	ns
**ANS-PNS/GoMe**	31° ± 0.3°	31° ± 0.6°	ns

ns: not significant. *p* value < 0.005.

**Table 3:** Descriptive statistics and statistical comparison between IF and BB groups at time T1.

	BB	IF	*p* Value
**FMA**	33° ± 1.2°	29 ± 0.8°	ns
**SN°GoGN**	43° ± 1.9°	37° ± 2.1°	ns
**Poccl ° PF**	15° ± 0.3°	10° ± 0.7°	ns
**N-ANS/N-SMe**	42% ± 1.6%	43% ± 1.2%	ns
**Sn°ANS-PNS**	10° ± 0.7°	9° ± 0.2°	ns
**Sn°Poccl**	24° ± 1.1°	20° ± 0.8°	ns
**SGo/N-Me**	58° ± 0.9°	60° ± 1.11°	ns
**ArGo/GoMe**	135° ± 2.8°	131° ± 2.1°	ns
**ANS-PNS/GoMe**	30° ± 1°	29° ± 0.7°	ns

ns: not significant. *p* value < 0.005.

**Table 4:** Descriptive statistics and statistical comparison between T0 and T1 differences by means of Student *t*-test for paired groups in BB group.

	T0	T1	*p* Value
**FMA**	34° ± 1.3°	33° ± 1.2°	ns
**SN°GoGN**	44° ± 2.1°	43° ± 1.9°	*
**Poccl ° PF**	16° ± 0.7°	15° ± 0.3°	*
**N-ANS/N-SMe**	42% ± 1.3%	42% ± 1.6%	*
**Sn°ANS-PNS**	9° ± 0.5°	10° ± 0.7°	*
**Sn°Poccl**	26° ± 1.4°	24° ± 1.1°	*
**SGo/N-Me**	57° ± 1.3 *	58° ± 0.9°	*
**ArGo/GoMe**	136° ± 2.5°	135° ± 2.8°	*
**ANS-PNS/GoMe**	31° ± 0.3°	30° ± 1°	*

ns: not significant. * *p* value < 0.005.

**Table 5:** Descriptive statistics and statistical comparison between T0 and T1 differences by means of Student *t*-test for paired groups in IF group.

	T0	T1	*p* Value
**FMA**	31° ± 0.9°	29 ± 0.8°	*
**SN°GoGN**	38° ± 1.9°	37° ± 2.1°	*
**Poccl ° PF**	11° ± 0.6°	10° ± 0.7°	ns
**N-ANS/N-SMe**	43% ± 1.7%	43% ± 1.2%	*
**Sn°ANS-PNS**	9° ± 0.3°	9° ± 0.2°	ns
**Sn°Poccl**	21° ± 1.2°	20° ± 0.8°	*
**SGo/N-Me**	59° ± 0.7°	60° ± 1.11°	*
**ArGo/GoMe**	135° ± 2.2°	131° ± 2.1°	*
**ANS-PNS/GoMe**	31° ± 0.6°	29° ± 0.7°	*

ns: not significant. * *p* value < 0.005.

## Data Availability

The raw data supporting the conclusions of this article will be made available by the authors on request.
